# Expression Patterns of Three UGT Genes in Different Chemotype Safflower Lines and under MeJA Stimulus Revealed Their Potential Role in Flavonoid Biosynthesis

**DOI:** 10.1371/journal.pone.0158159

**Published:** 2016-07-08

**Authors:** Dan-Dan Guo, Fei Liu, Yan-Hua Tu, Bei-Xuan He, Yue Gao, Mei-Li Guo

**Affiliations:** Department of Pharmacognosy, College of Pharmacy, Second Military Medical University, 200433, Shanghai, China; Wuhan Botanical Garden of Chinese Academy of Sciences, CHINA

## Abstract

Safflower (*Carthamus tinctorius* L.) has received a significant amount of attention as a medicinal plant in China. Flavonoids are the dominant active medical compounds. UDP-glycosyltransferase plays an essential role in the biosynthesis and storage of flavonoids in safflower. In this study, 45 UGT unigenes were screened from our transcriptomic database of safflower. Among them, 27 UGT unigenes were predicted to own a complete open reading frame with various pI and Mw. The phylogenetic tree showed that *Ct*UGT3 and *Ct*UGT16 were classified under the UGT71 subfamily involved in metabolite process, whereas *Ct*UGT25 has high identities with *Po*UGT both catalyzing the glycosylation of flavonoids and belonging to the UGT90 subfamily. cDNA microarray exhibited that the three UGT genes displayed temporal difference in two chemotype safflower lines. To functionally characterize UGT in safflower, *Ct*UGT3, *Ct*UGT16 and *Ct*UGT25 were cloned and analyzed. Subcellular localization suggested that the three UGTs might be located in the cell cytoplasm and chloroplast. The expression pattern showed that the three UGTs were all suppressed in two lines responsive to methyl jasmonate induction. The co-expression relation of expression pattern and metabolite accumulation demonstrated that *Ct*UGT3 and *Ct*UGT25 were positively related to kaempferol-3-*O*-β-D-glucoside and *Ct*UGT16 was positively related to quercetin-3-*O*-β-D-glucoside in yellow line, whereas *Ct*UGT3 and *Ct*UGT25 were positively related to quercetin-3-*O*-β-D-glucoside in white line. This study indicates that the three *Ct*UGTs play a significant and multiple role in flavonoids biosynthesis with presenting different functional characterization in two safflower lines.

## Introduction

Safflower (*Carthamus tinctorius* L.) is cultivated mainly for medicinal use, with its dried tubular flowers being the medicinal part and its seeds being commonly consumed as vegetable oil in many countries due to their abundant unsaturated fatty acid, oleic acid and α-linoleic acid [1]. Flavonoid compounds, quinochalcone glycosides [hydrosafflower yellow A (HSYA), carthamin, tinctorimine, cartorimin]. and flavonol glycosides (kaempferol glucosides and quercetin glucosides) are considered as the characteristic and active constituents in safflower [2] and pose a wide spectrum of biological and pharmacological effects, such as cerebrovascular and cardiovascular protective activities [3–5].

With the boom of the large-scale transcriptomic analysis, various deep sequencing techniques were employed in plants, such as in *Lonicera japonica Thunb*. [6], *Lilium regale* [7] and *Panax ginseng* [8]. The transcriptome of safflower was obtained to explore the gene family involved in the biosynthesis of flavonoids, such as phenylalanine ammonia-lyase (PAL), cinnamate-4-hydroxylase (C4H), chalcone synthase (CHS), chalcone isomerase (CHI), flavonone 3-hydroxylase (F3H) and flavonoid UDP-glycosyltransferase (UGT).

Many researches have demonstrated that UGT plays an essential role in the biosynthesis of secondary metabolites in plants [9]. UGT transfers nucleotide- diphosphate-activated sugars to low molecular weight (Mw) substrates. Intrinsic sugar donor in plants contains UDP-glucose, UDP-galactose, UDP-rhamnose, and UDP-glucuronic acid [10]. Sugar moieties, as part of many bioactive natural products, have significant effects on the physiological activity, selectivity, and other pharmacological properties [11,12]. The UGT family belongs to group 1 of a larger family of glycosyltransferases that have a similar protein structure (GT-B Rossmann-fold) [10]. The present UGT homepage (http:www.flinders.edu.au/medi-cine/sites/clinical-pharmacology/ugt-homepage.cfm) consists of a large list of UGT families, subfamilies and hundreds of approved UGTs, where families 71 to 100 are for plants [13]. The UGT superfamily is characterized by a common protein structure and a well-conserved sequence of 44 amino acids (called a PSPG box) responsible for binding the UDP moiety of the sugar donor [14–16]. Glycosylation is a prominent modification reaction [17]; therefore, the glycosylation process of flavonoids in safflower is important to biosynthesize the important active flavonoid compounds.

In the past few years, studies on plant glycosyltransferase have revealed that dramatically varied glycosyltransferases are involved in plant secondary metabolism [16,18], such as *Arabidopsis thaliana*, *cereals*, *Catharanthus roseus*, *rice*, *Crocus sativus*, *and Litchi chinensis* [19–26]. However, as an important medicinal plant with glycosylated flavonoids [27,28], the glycosylation process is largely unknown and rarely reported. Only one UGT (UGT73AE1) has been explored, but there is no biological view to explain the link between the gene and metabolic pathway evolution [29]. Here, we report the characterization of three UGTs through their in vitro and in vivo expression as well as their metabolite analysis response to methyl jasmonate (MeJA). These findings partly demonstrate the potential functionality of UGTs in the glycosylation of the flavonoids metabolism pathway in safflower.

## Materials and Methods

### Plant materials

**Fig 1:**
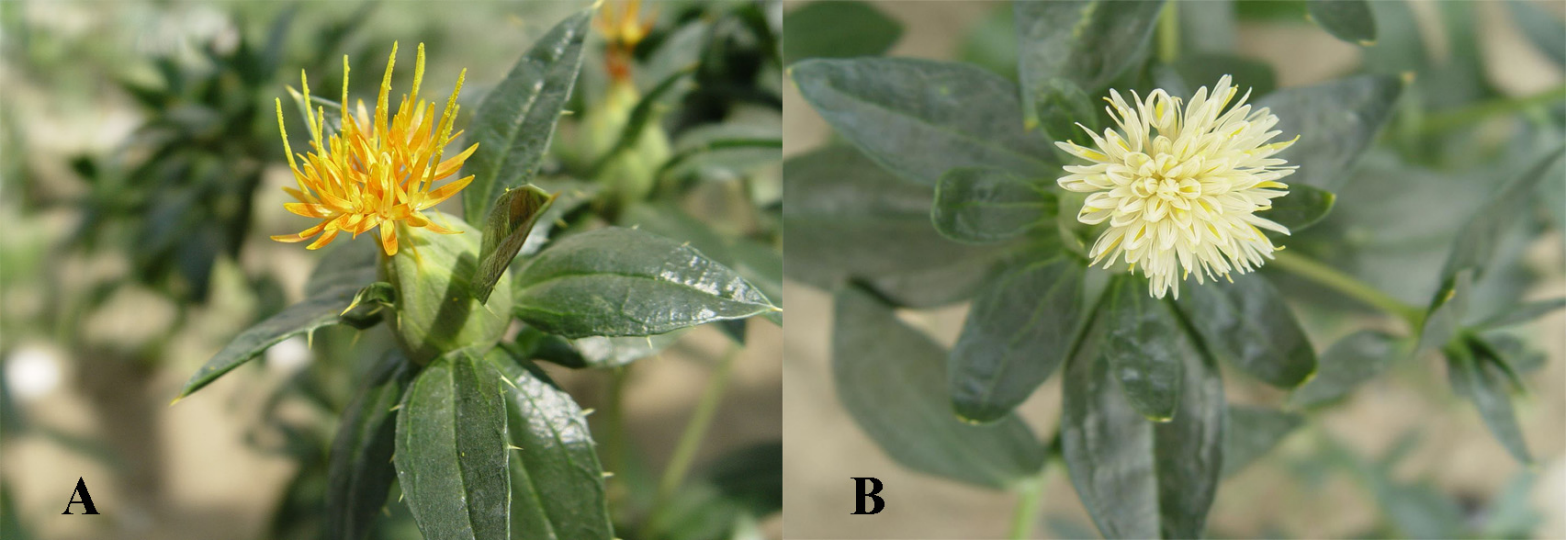
Two lines of safflower. (A) yellow flower line. (B) white flower line.

Two lines of safflower (ZHH0119 with yellow flower line and XHH007 with white flower line; Fig 1) were grown in the greenhouse of the Second Military Medical University (Shanghai City, China). The ZHH0119 line, which has orange-yellow floret, is a major source of quinochalcones and flavonols, whereas the XHH007 line with white floret, mainly contains flavonols without quinochalcones. The plants were cultivated at a mean temperature of 25°C with circadian rhythm of 16 h-light/8 h-darkness.

### Transcriptome data analysis and multiple sequence alignment of *Ct*UGT genes

To reduce gene copy variation on the transcription level, normalized cDNA libraries were constructed with the total RNA of equivalent different developmental stage flower samples. Then, safflower transcriptome sequencing was performed to find more genes involved in the biosynthetic pathway of active components. Gene annotations were predicted by BLASTx and BLASTn search homologs in the Nr and Nt database. UGTs were selected from annotated genes. The open reading frame (ORF) prediction was carried out and amino acids were deduced in ORF Finder. The theoretical isoelectric point (pI) and Mw were predicted using the Compute pI/Mw tool on the ExPASy server (http://web.expasy.org/compute_pi/). The multiple sequence alignment of the UGTs was performed using ClustalX version 2.0 [30]. Phylogenetic trees were constructed using MEGA 5.0 with the neighbor-joining method. Bootstrap test was replicated 1000 times [31].

### Agilent cDNA expression profile microarray

Using cDNA expression profile microarray, many differently expressed genes were identified. Signal values were normalized with log2. Hot map for expression signal intensity and hierarchical clustering (HCL) were conducted by MeV [32], whereas stage I of the two lines was treated as the control group. All samples with three biological repeats were carried out.

### Cloning of CtUGT full-length cDNA

Total RNA was isolated with the RNeasy Plant Mini Kit (Qiagen, Germany) according to the manufacturer’s protocol. The 3′- and 5′- cDNA libraries of safflower were constructed using the Clontech Smart^TM^ Rapid Amplification of cDNA Ends (RACE) cDNA amplification kit (Clontech, USA). Then, RACE was carried out. Based on the sequences of the 3′- and 5′-RACE products, the primer were designed to clone the full length of genes. Polymerase chain reaction (PCR) was performed using cDNA (TransScript® One-Step gDNA Removal and cDNA Synthesis Super Mix; TransGen Biotech, Beijing, China) as a template and with KOD-Plus-Neo polymerase (Toyobo, Japan) under the following conditions: 30 cycles of 10 s denaturation at 94°C, 30 s annealing at 58°C, and 1 min amplification at 72°C. The PCR products were gel-purified (QIAquick® Gel Extraction Kit; Qiagen) and cloned into the PMD19T vector (TaKaRa, Japan). Using blue-white spot screening, the recombinant plasmids were recovered from *Escherichia coli* DH5α cells using QIAquick® Spin Plasmid Mini-prep kit (Qiagen) and both strands were sequenced (MeiJi, China).

### Bioinformatics of three *Ct*UGTs

The obtained full-length cDNA sequence and deduced protein were analyzed using the following websites: www.ncbi.nlm.nih.gov and www.expasy.org. The tertiary structure of *Ct*UGT3, *Ct*UGT16, and *Ct*UGT25 was predicted on www.swissmodel.expasy.org. Protein subcellular localization was predicted in WoLF PSORT (advanced computational tool for protein subcellular location prediction).

### Induction and quantitative real-time PCR of MeJA-treated safflower petals

MeJA (100μM; Sigma, USA) solution was sprayed onto petals of safflower that opened on the first day. The petals were then enclosed in plastic bags. In the control group, the petals were sprayed with the same solution but without MeJA and then covered with plastic bags. After 0, 3, 6, and 12 h of treatment, the plastic bags were removed, and at least three samples of flowers at four time points were collected; these samples were frozen immediately in liquid nitrogen and stored in freezers at -80°C.

cDNAs were synthesized with 1 μg RNA according to the manufacturer’s instructions of TransScript® One-Step gDNA Removal and cDNA Synthesis SuperMix (TransGen Biotech). Q-PCR [33] was performed according to the instructions of the SYBR Green Realtime Master Mix kit (Toyobo) with the ABI 7500 system (ABI, USA). The specific primers used for quantification were designed using Primer 5. PCR conditions comprised an initial holding at 95°C for 3min. the cycle stage of the PCR program consists of 95°C for 10 s and 58°C for 20 s and 72°C for 35 s for 40 cycles. Standard deviations were calculated from three PCR replicates. The specificity of amplification was assessed by dissociation curve analysis, and the relative abundance of genes was determined using the comparative Ct method and normalized by that for 60S.

### Metabolite analysis of MeJA treated by UPLC-QTOF/MS

Safflower petal samples were dried at 50°C to constant weight and ground into powder. Subsequently, an aliquot of 10 mg samples was soaked overnight and extracted for 40 min sonication with 60% methanol under sealed conditions. Then, the extract was filtered through a 0.20 μm microporous membrane for analysis. The metabolites were identified using Agilent Technologies 6538 UHD Accurate Mass Q-TOF LC/MS (Agilent Technologies 1290 Infinity). Waters XSELECT HSS T3 (100×2.1 mm, 2.5 μm); mobile phase A, 0.1% methanoic acid; mobile phase B, acetonitrile with 0.1 formic acid; flow rate, 0.4 ml/min; column temperature, 40°C; gradient elution; 0-2min, A:B = 95:5, 2–4 min, A:B = 80:20, 4–6 min, A:B = 79:21, 6–9 min, A:B = 74:26, 9–11 min, A:B = 60:40, 11–15 min, A:B = 20:80, 15–17 min, A:B = 5:95, and 17–19 min, A:B = 5:95; injection volume: 4 μl.

Mass spectrometer was performed and positive ion mode was used for the quantification. Mass acquisition range: 100–1000; gas temperature, 350°C; gas flow, 11 L/min; nebulizer, 45 psi; Vcap, 4000V; fragmentor, 120V; skimmer, 60v; octopoleRFPeaK, 750v; reference masses, m/z 121.0509 and 922.0098. 17 compounds were dealed with target compound and analyzed. 12 standard chemical compounds were purchased from Sigma-Aldrich (St. Louis, MO).

## Results

### Transcriptome data analysis, multiple sequence alignment, and phylogenetic analysis

**Fig 2:**
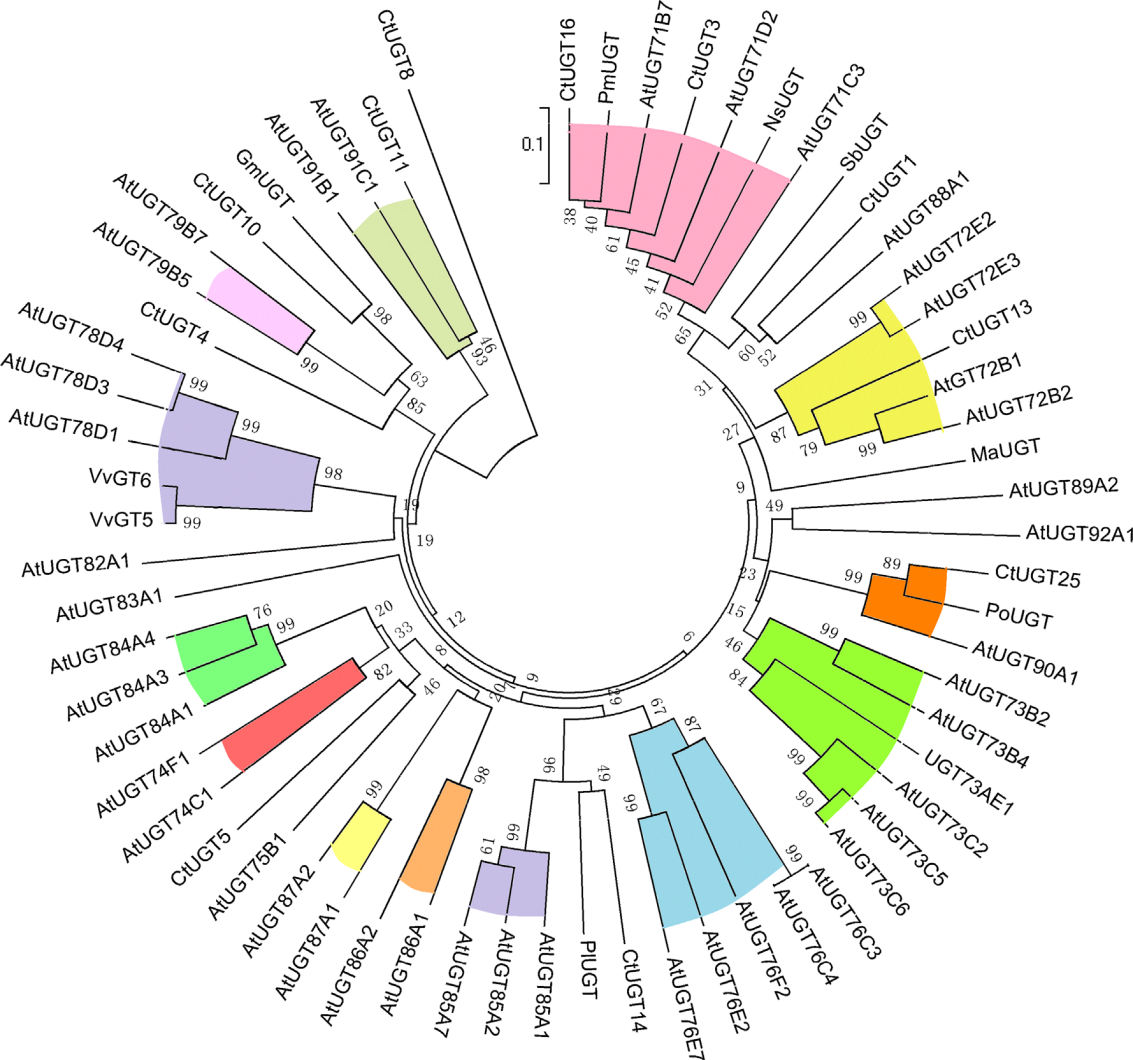
Phylogenic tree of UGTs from *C*.*tinctorius*. The phylogenetic relationship of UGTs from *C*.*tinctorius* and other plants were analyzed with ClustalX and MEGA5 using the amino acid sequences.

**Fig 3:**
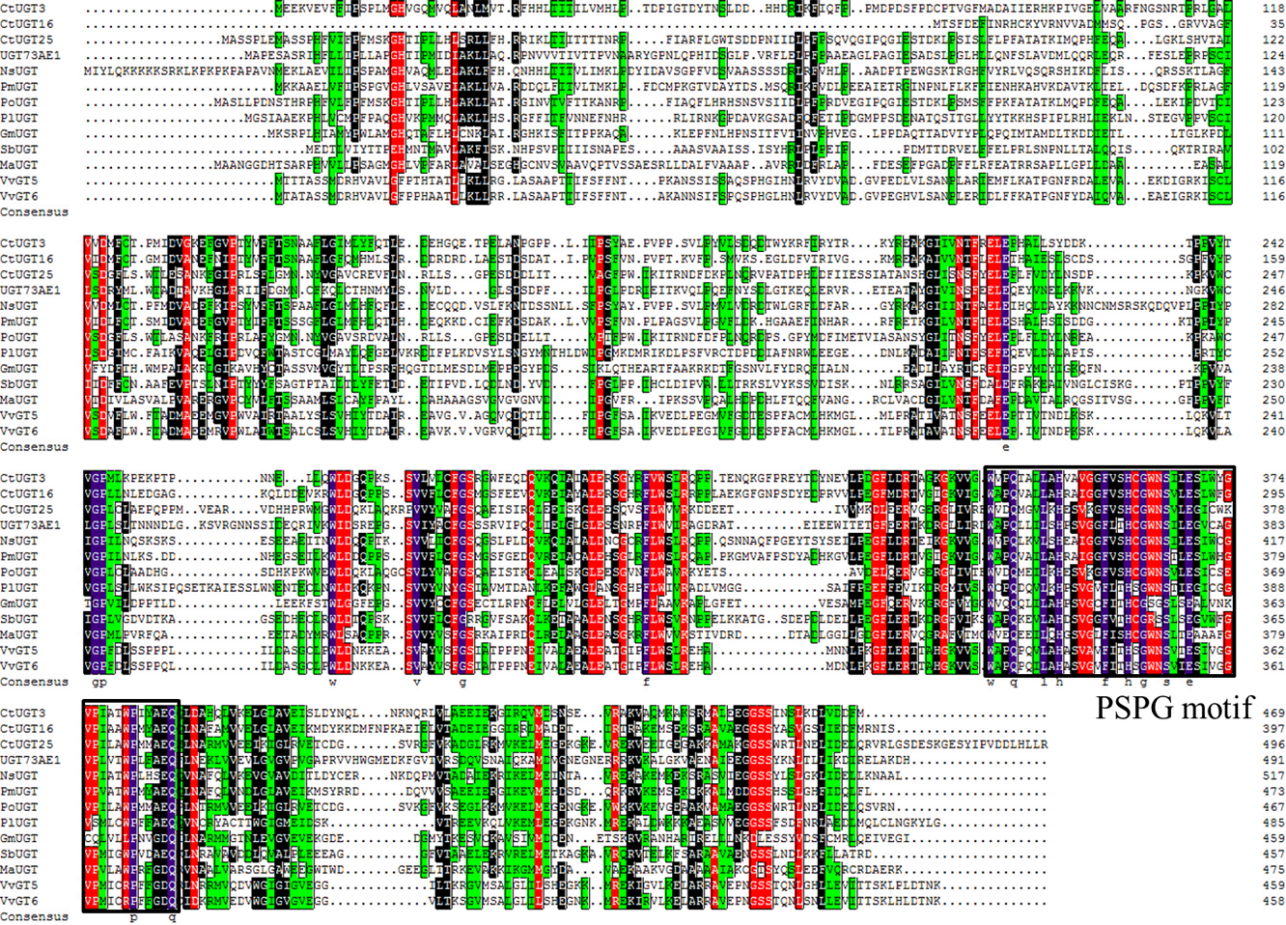
A multiple alignment of the amino acid sequences of three *Ct*UGT from *C*.*tinctorius* and other plant UGTs. *Vv*GT5 (GenBank accession number BAI22846.1 from *Vitis vinifera*), *Vv*GT6 (BAI22847.1 from *V*. *vinifera*), *Sb*UGT (KP183919.1 from *Scutellaria baicalensis*), *Ma*UGT (A0A096SRM5.1 from *Maize*), *Pl*UGT (AFI71901.1 from *Paeonia lactiflora*), *Gm*UGT (BAR88078.1 from *Glycine max*), *Po*UGT (ACB56926.1 from *Pilosella officinarum*), *Ns*UGT (XP_009766439.1 from *Nicotiana sylvestris*), *Pm*UGT (XP_008229635.1, from *Prunus mume*), UGT73AE1 (AJT58578.1 from *C*. *tinctorius*). All of them are flavonoid glycosyltransferases. Identical amino acids are shown in white on a purple background. The black box indicates the conserved region of plant secondary product glycosyltransferases (PSPG motif).

In order to obtain the maximized coverage of genes, a mixed RNA sample from different stages of petals was applied to construct a cDNA library. From the annotation of safflower transcriptome, 45 unigenes coding UGTs were identified (S1 Table). In general, plants contain a high copy number of UGT genes, among which 121 genes have been identified in *A*. *thaliana* [34] and 165 genes in *Medicago truncatula* [35]. Using an ORF search, 27 unigenes in safflower were predicted to have full-length cDNA sequence. And their pI and Mw are shown in Table 1. Their pI is from 5.5 to 7.5, but Mw varies greatly. 27 *Ct*UGT genes were translated into proteins in S2 Table. Based on *Ct*UGTs, *At*UGTs and UGTs from other plant species (Fig 2), the phylogenetic tree was constructed. Our results showed that *Ct*UGT3 and *Ct*UGT16 were classified under the UGT71 subfamily involved in metabolite process, whereas *Ct*UGT25 has high identities with *Po*UGT (GenBank accession number ACB56926.1 from *Pilosella officinarum*) both catalyzing the glycosylation of flavonoids and belonging to the UGT90 subfamily. Given the high identities of the reported flavonoid GTs (Fig 3) in multiple sequence alignment, three *Ct*UGT genes can be tentatively assigned as a flavonoid glycosyltransferase in *C*. *tinctorius*. Therefore, to functionally characterize UGT in safflower, *Ct*UGT3, *Ct*UGT16, and *Ct*UGT25 were cloned and analyzed.

**Table 1:** Identification of UGT genes in safflower by transcriptome sequencing.

Gene name	unigene	CDS(bp)	ORF(aa)	PI	Mw(KDa)
***Ct*UGT1**	Contig837	1416	471	5.51	51.99
***Ct*UGT2**	Contig1071	783	260	6.6	29.16
***Ct*UGT3**	Contig1097	1410	469	5.31	52.95
***Ct*UGT4**	Contig1841	1368	455	6.34	51.20
***Ct*UGT5**	Contig2153	729	242	5.3	26.76
***Ct*UGT6**	Contig2267	267	88	5.47	10.20
***Ct*UGT7**	Contig2457	741	246	5.33	26.80
***Ct*UGT8**	Contig2737	1053	350	7.18	40.41
***Ct*UGT9**	Contig2789	300	99	5.21	10.84
***Ct*UGT10**	Contig2794	1380	459	5.91	51.39
***Ct*UGT11**	Contig3171	798	265	4.97	29.53
***Ct*UGT12**	Contig3742	696	231	7.06	26.72
***Ct*UGT13**	Contig3776	1071	356	5.95	39.46
***Ct*UGT14**	Contig3800	1440	479	5.55	53.72
***Ct*UGT15**	Contig3889	234	77	7.85	8.51
***Ct*UGT16**	A09-CS0907125834-R044-9-M13F(-20).ab1	1194	397	4.92	43.96
***Ct*UGT17**	B05.CS090410701_7179.A2-80.M13F(-20).ab1	399	132	6.13	15.29
***Ct*UGT18**	B11.CS090904_3135.S005-23.M13F(-20).ab1	690	229	6.05	25.79
***Ct*UGT19**	B12-CS0909027-6681-R117-24-M13F(-20).ab1	270	89	9.9	9.93
***Ct*UGT20**	CS090410709_3045.A4-46.M13F(-20)_E03.ab1	405	134	8.61	14.87
***Ct*UGT21**	CS090415301_3195.A9-96.M13F(-20)_A01.ab1	309	102	5.42	11.37
***Ct*UGT22**	CS090423305_3594.E4-95.M13F(-20)_A02.ab1	309	102	4.95	11.36
***Ct*UGT23**	CS090519307_3752.L4-53.M13F_D08.ab1	642	213	4.9	24.03
***Ct*UGT24**	CS090523704_7446.Q3-47.M13F_E02.ab1	195	64	9.35	7.35
***Ct*UGT25**	D07-CS090907_6447-R58-043-M13F(-20).ab1	1497	498	5.84	56.01
***Ct*UGT26**	G12.CS090410702_7212.A3-13.M13F(-20).ab1	159	52	6.57	5.95
***Ct*UGT27**	H12-CS091005-6779-R169-96-M13F(-20).ab1	291	96	7.86	11.19

### cDNA microarray expression analysis

**Fig 4:**
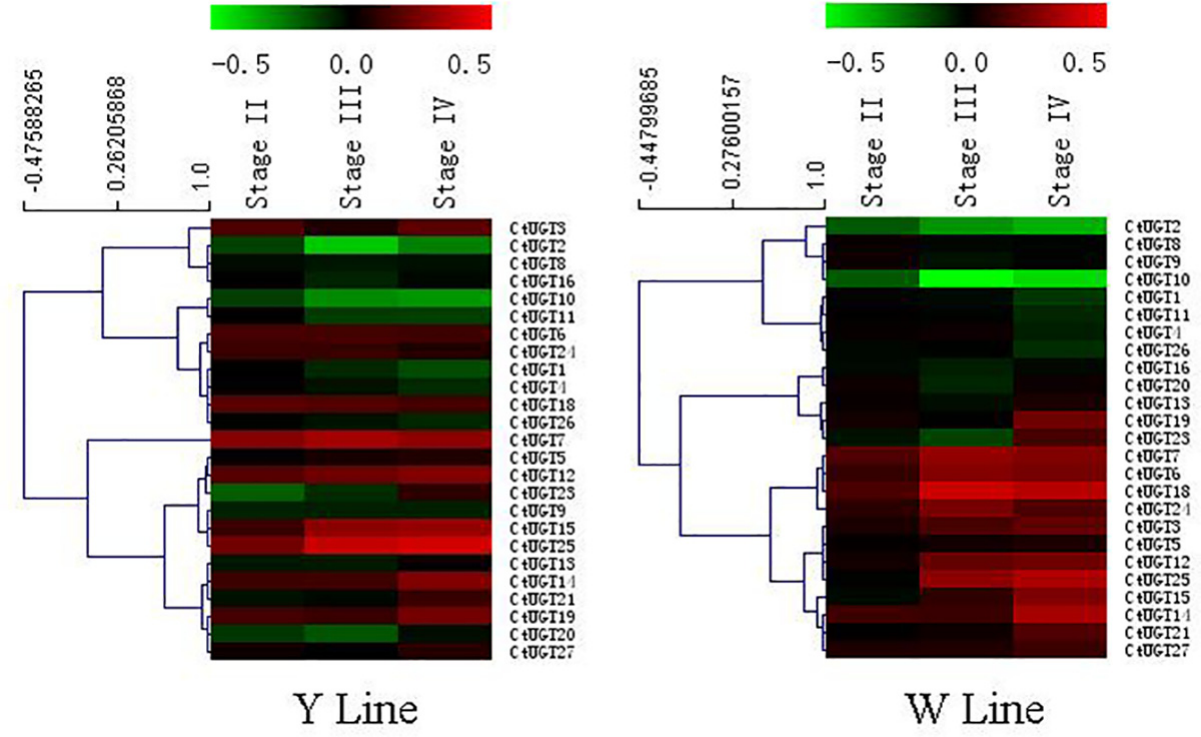
Microarray expression abundance and HCL cluster at different stages of petals for the yellow and white line. (I) 10 days before blooming. (II) 5 days before blooming. (III) blooming days. (IV) 2 days after blooming.

From microarray data, differential genes were determined (p≤0.05) by comparing two groups in yellow and white lines respectively. Because flags were 3A (not obvious between signal and background) found in microarray data, two UGTs (UGT17 and UGT22) were removed. Hot map revealed that 25 UGTs have different expression at different stages of yellow and white lines. The colour key is from -0.5 to 0.5. Obviously, the expression values of all 25 UGTs present different expression models at different developmental stages of yellow and white line (Fig 4). Both up-regulation genes from two lines contained 11 UGT genes (*Ct*UGT5, *Ct*UGT7, *Ct*UGT12, *Ct*UGT14, *Ct*UGT15, *Ct*UGT19, *Ct*UGT21, *Ct*UGT23, *Ct*UGT24, *Ct*UGT25, and *Ct*UGT27, whereas the other 6 UGT genes displayed down-regulation (*Ct*UGT1, *Ct*UGT2, *Ct*UGT4, *Ct*UGT11, *Ct*UGT17, and *Ct*UGT26). Microarray data demonstrated that *Ct*UGT3, *Ct*UGT16 and *Ct*UGT25 with close genetic relationship showed different expression models in two safflower lines.

### Cloning and sequence analysis of *Ct*UGT genes

To confirm that differential UGT genes were indeed expressed and analyze the relation between gene expression profile and metabolite accumulation in two safflower lines, *Ct*UGT3, *Ct*UGT16 and *Ct*UGT25 genes were chosen for qRT-PCR analysis.

**Fig 5:**
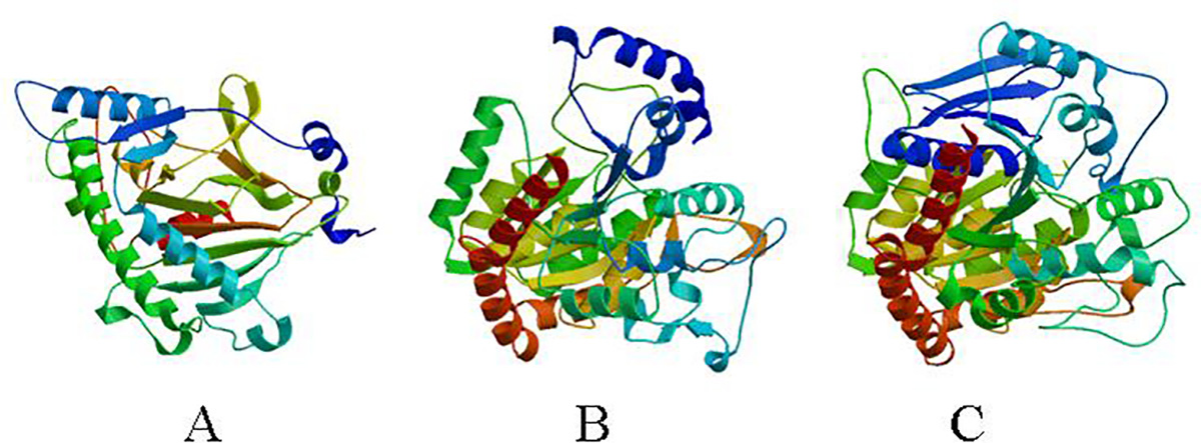
Predicted protein tertiary structure of UGT from safflower. (A) *Ct*UGT3. (B) *Ct*UGT16. (C) *Ct*UGT25.

The full-length cDNA of *Ct*UGT genes were isolated from safflower and their proteins were given the names *Ct*UGT3, *Ct*UGT16, *Ct*UGT25 (GenBank accession numbers KT947113, KT947114, and KT947116), and the primers are shown in Table 2. The sequences of PCR products were consistent with the predicted sequence. The 3D structures of three UGT proteins were predicted by SWISS model (beta.swissmodel.expasy.org) (Fig 5). The nucleotide sequence and the deduced amino acid sequence of three UGTs were predicted (linux1.softberry.com). In WoLF PSORT (advanced computational tool for protein subcellular localization prediction), protein subcellular localization predicted *Ct*UGT3 has 14 nearest neighbors, including 10 cyto, 2 chlo, and 1 nucl. *Ct*UGT16 has 14 nearest neighbors, 11 cyto and 3 E.R.. *Ct*U*GT25* has 14 nearest neighbors, containing 12.5 chlo and 7.5 chlo_mito. Signal P4.1 Server predicted three UGTs have no signal peptide (SP). Protscale (http://expasy.org/cgibin/protscale.pl) predicted three UGTs from hydrophobicity based on Kyte-Doolittle. These results indicated that the three UGTs may not take part in protein transport and act in the catalytic activity of the enzyme in the cell cytoplasm and chloroplast.

**Table 2:** Primer of UGTs.

Gene name	Primer sequence
***C*tUGT3-F (for full-length)**	CTTGTCGACTAGTTCCATGAAATCG
***Ct*UGT3-R (for full-length)**	AAGAGCCCATGGAGGAGAAAGTAG
***Ct*UGT16-F (for full-length)**	AAGCGGCCGCTTGATGAGATGTTC
***C*tUGT16-R (for full-length)**	AATCCATGGCCCATGACTTCTTTCG
***Ct*UGT25-F (for full-length)**	TCTAGCCATGGCTTCCCTCACCTCATT
***C*tUGT25-R (for full-length)**	ATAGGATTCTTCGATTCTTAGGAGGTGTA
**CtUGT3-F (for qRT-PCR)**	CCAAGAGACCATCAGACAA
**CtUGT3-R (for qRT-PCR)**	GGCACAGTTCCAATACCA
**CtUGT16-F (for qRT-PCR)**	GGAAGAGGAGTATGATGA
**CtUGT16-R (for qRT-PCR)**	AATGGCAGTTGAGATTAC
**CtUGT25-F (for qRT-PCR)**	CTACACCAACCTCAATCG
**CtUGT25-R (for qRT-PCR)**	AGTCACATCCACATTCAAG

### Temporal expression pattern of *Ct*UGT genes in MeJA- treated *C*. *tinctorius* inflorescence

As a well-known exogenous induced factor, MeJA is reported to have taken part in many plant processes, ranging from plant defense to growth and development [36]. MeJA is of particular interest in plant cell engineering for producing bioactive compounds [37,38]. Therefore, MeJA plays a critical regulatory role in the biosynthesis of various active secondary metabolites. To investigate how the flavonoid biosynthetic pathways respond to MeJA, the expression pattern of the relative genes was detected in MeJA-treated *C*.*tinctorius* inflorescence.

**Fig 6:**
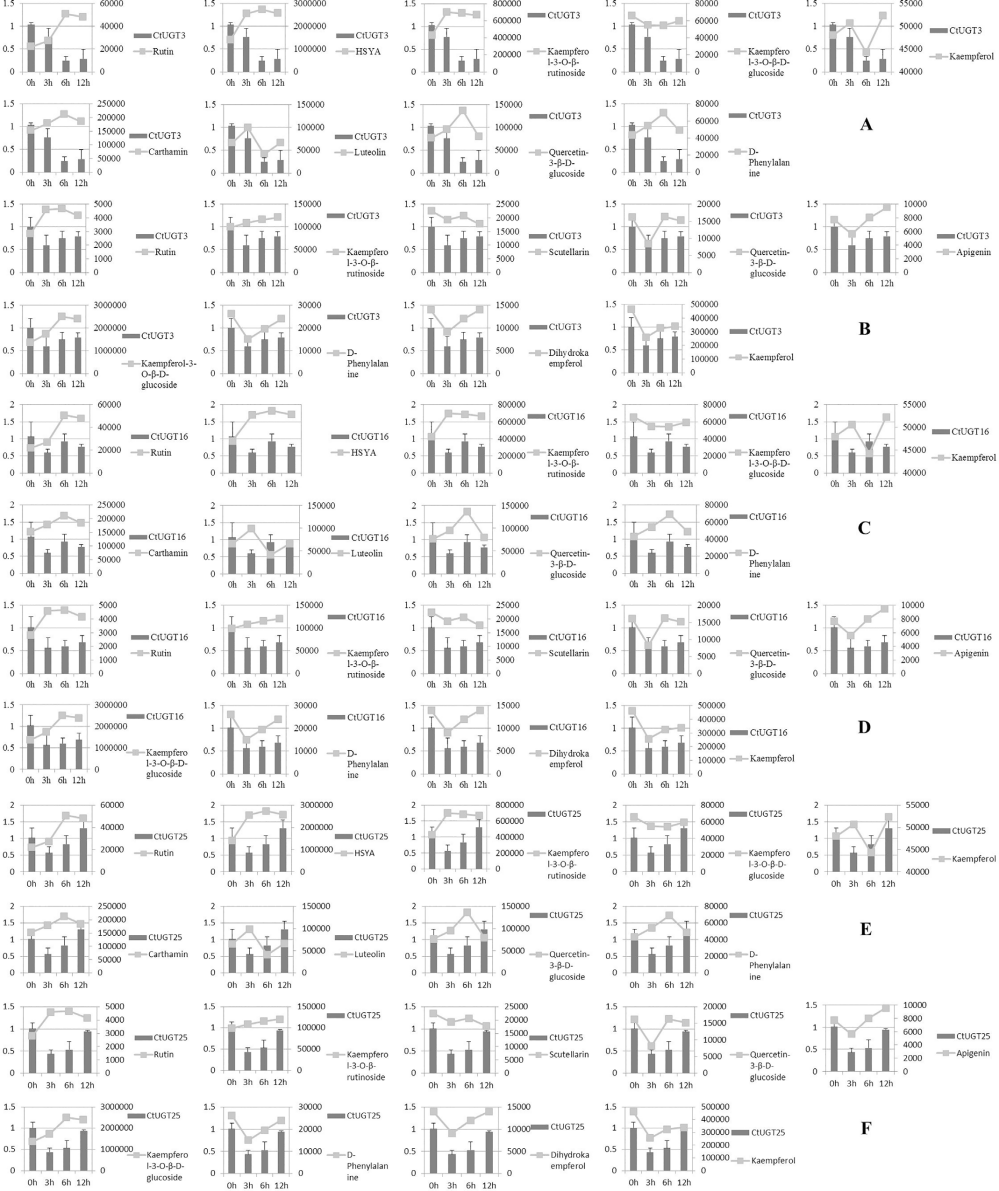
Expression pattern of three UGT genes and relative content changes of flavonoid compounds in *C*.*tinctorius* treated with MeJA for 0, 3, 6, and 12 h. Primary axis: relative expression level. Secondary axis: relative content of compounds. Expression levels were quantified by quantitative PCR. The level of each gene is relative to that of 60 s reference. Each data point is the average of three biological repeats. Error bars indicate SD. A, C, and E are yellow line. B, D and F are white line. A and B are *Ct*UGT3, C and D are *Ct*UGT16, E and F are *Ct*U*GT25*.

The expression pattern of *Ct*UGTs was investigated by extracting total RNA from inflorescence. Real-time PCR primers were designed in Table 2. As shown in Fig 6, *Ct*UGT transcript levels were examined and varied. The highest expression level was observed at oh treatment generally. These suggested that three UGTs were all suppressed in response to MeJA-induction. In two safflower lines, we found that *Ct*UGT3 (0.2457 and 0.2868; Fig 6A and 6B) descended more in yellow line over MeJA treatment time compared to white line. *Ct*UGT16 (0.5660 and 0.6018; Fig 6C and 6D) and *Ct*UGT25 (0.4360 and 0.5315; Fig 6E and 6F) were inhibited obviously at 3 and 6h in white line. Expression analysis in petals during flower development indicated that *Ct*UGT3 may take part in glycosylation in yellow safflower line easily than in white line, and *Ct*UGT16 and *Ct*UGT25 may tend to regulate flavonoid glycosylation in white line.

### Analysis of flavonoid compound accumulation

To discuss the correlation of genes and related metabolites by MeJA-induction, the accumulation pattern of flavonoid compounds has been examined using UPLC-QTOF/MS in our laboratory. Using MASS Hunter workstation, we extracted and searched for target components after MeJA treatment for 3, 6 and 12 h compared to control (S3 Table**)**. 12 flavonoid compounds were detected by our team, including rutin, HSYA, kaempferol-3-*O*-β-D-rutinoside, kaempferol-3-*O*-β-D-glucoside, carthamin, luteolin, quercetin-3-*O*-β-D-glucoside (S3 Table). These compounds were stimulated under MeJA treatment and possess different accumulation models. In yellow line, rutin, HSYA, kaempferol-3-*O*-β-D-rutinoside, carthamin, and quercetin-3-*O*-β-D-glucoside were induced, and raised constantly at 0, 3, and 6h, whereas only kaempferol-3-*O*-β-D-glucoside was inhibited. Rather, rutin, kaempferol-3-*O*-β-rutinoside and kaempferol-3-*O*-β-D-glucoside were raised, scutellarin and quercetin-3-*O*-β-D-glucoside were inhibited after treatment in white line. This indicates that accumulation pattern of secondary products varied in different chemotype safflower lines.

### Co-expression analysis of expression pattern and metabolite accumulation

The co-expression analysis of “gene- metabolites” is displayed in Fig 6. Results indicated that *Ct*UGT3 and *Ct*UGT25 were positively related to kaempferol-3-*O*-β-D-glucoside and *Ct*UGT16 was positively related to quercetin-3-*O*-β-D-glucoside in yellow line, whereas *Ct*UGT3 and *Ct*UGT25 were positively related to quercetin-3-*O*-β-D-glucoside in white line (Table 3). This suggests that *Ct*UGT3 and *Ct*UGT25 may take part in regulating flavonol-3-*O*-glucoside biosynthesis in two line, whereas *Ct*UGT16 regulats flavonol-3-*O*-glucoside biosynthesis in yellow line. Meanwhile, we found that three *Ct*UGT genes are irrelevant to chalcone glycosides biosynthesis in two safflower lines. Our results indicated that the three *Ct*UGTs are the potential flavonol glycosyltransferases presenting different functional characterization of flavonoid biosynthesis in two safflower lines.

**Table 3:** The full set of correlation coefficients between UGTs and flavone glycoside compounds.

Pearson r	*Ct*UGT3-Y	*Ct*UGT3-W	*Ct*UGT16-Y	*Ct*UGT16-W	*Ct*UGT25-Y	*Ct*UGT25-W
**Rutin-Y**	-0.6069		0.4374		0.3254	
**Hydroxysafflor yellow A-Y**	-0.5391		0.0350		-0.2581	
**Kaempferol-3-O-β-rutinoside-Y**	-0.5195		-0.0665		-0.3455	
**Kaempferol-3-O-β-D-glucoside-Y**	0.8776		-0.1470		0.6010	
**Carthamin-Y**	-0.2942		0.4945		-0.1451	
**Luteolin-Y**	-0.1252		-0.9595		-0.3905	
**Quercetin-3-O-β-D-glucoside -Y**	0.2612		0.6969		-0.4436	
**D-Phenylalanine-Y**	0.0906		0.6000		-0.4254	
**Kaempferol-Y**	-0.6706		-0.9258		0.1845	
**Rutin-W**		-0.8777		-0.9899		-0.7643
**Kaempferol-3-O-β-rutinoside-W**		-0.4260		-0.6677		-0.1617
**Scutellarin-W**		0.5751		0.6168		0.1300
**Quercetin 3-O-β-D-glucoside-W**		0.8065		0.5452		0.6891
**Kaempferol-3-O-β-D-glucoside-W**		-0.3723		-0.6639		-0.2323
**D-Phenylalanine-W**		0.9425		0.8307		0.9654
**Dihydrokaempferol-W**		0.8765		0.6988		0.9189
**Kaempferol-W**		0.9971		0.9508		0.8468
**Apigenin-W**		0.5927		0.3256		0.7288

## Discussion

Plant transcriptome sequences appear in increasing numbers, such as in *maize* [39], *Triticum aestivum* L. [40], *Cassia angustifolia Vahl* [41] and *Piper nigrum* [42]. Due to the desire for a deep understanding of the key genes of active metabolite biosynthetic processes, the complete transcriptome of *C*.*tinctorius* was sequenced and analyzed in our lab.

Considering the significance of UGT in the biosynthesis of plant secondary metabolites [43,44], 45 *Ct*UGT unigenes were identified and characterized by BLASTn and BLASTx in Nr database from our transcriptomic database in safflower florets. The phylogenetic tree showed that *Ct*UGT3 and *Ct*UGT16 were classified under the UGT71 subfamily involved in metabolite process, whereas *Ct*UGT25 has high identities with *Po*UGT (GenBank accession number ACB56926.1 from *P*. *officinarum*) both catalyzing the glycosylation of flavonoids and belonging to the UGT90 subfamily. Given the high identities to the reported flavonoid UGTs in multiple sequence alignment, the three *Ct*UGT genes can be assigned as a flavonoid glycosyltransferases in *C*.*tinctorius*. The expression profile of three *Ct*UGTs responsive to MeJA- induction showed high expression level with a rapid response at the moment of sprayed. Then, three *Ct*UGTs have down-regulated trends over the time of MeJA- induction. The co-expression analysis results indicated that three *Ct*UGTs have various regulation models with flavonoid glycosides in two safflower lines. *Ct*UGT3 and *Ct*UGT25 present high positive regulation on kaempferol-3-*O*-β-D-glucoside and quercetin-3-*O*-β-D-glucoside in two lines. *Ct*UGT16 showed high positive regulation on quercetin-3-*O*-β-D-glucoside in yellow line. Also, *Ct*UGT3 and *Ct*UGT25 displayed up-regulated pattern in both yellow and white lines during the development of flower from the microarray data. Our metabolite data (unpublished) demonstrated that accumulation of kaempferol-3-*O*-β-D-glucoside and quercetin-3-*O*-β-D-glucoside are up-regulated with flower elongation, which is coincident with the co-expression analysis result by MeJA-treatment. Therefore, *Ct*UGT3 and *Ct*UGT25 may not only regulate flavonol biosynthesis but also be involved in flower development. Additionally, three *Ct*UGTs showed no link to quinochalcones such as HSYA and carthamin in yellow line, indicating these three *Ct*UGTs only affected flavonol glycosides biosynthesis and have no influence on quinochalcones biosynthesis. To sum up, our results reveal that the *Ct*UGTs present different functional characterization of flavonoid biosynthesis and flower development in different safflower lines. Although further clues need to be understood the physiological roles of these genes, the obtained findings suggest that *Ct*UGT3, *Ct*UGT16 and *Ct*UGT25 genes are not only involved in the process of flavonol glucosides biosynthesis but also in flower development with presenting different functional characterization of flavonoid biosynthesis in different chemotype safflower lines. Our work may help elucidate the roles of glycosyltransferase in flavonoid biosynthesis pathway. Next, we are trying to further identify more functional UGT genes in safflower.

## Supporting Information

S1 Table List of putative UDP-glycosyltransferases in *C*.*tinctorius*. (XLSX)

S2 Table UGTs proteins sequence with complete CDS. (XLSX)

S3 Table Flavonoids compounds accumulation changes (peak area/weight) by MeJA-induced changes in *C*.*tinctorius* two line. (XLSX)
